# Behavioral and ERP measures of attentional bias to threat in the dot-probe task: poor reliability and lack of correlation with anxiety

**DOI:** 10.3389/fpsyg.2014.01368

**Published:** 2014-12-04

**Authors:** Emily S. Kappenman, Jaclyn L. Farrens, Steven J. Luck, Greg Hajcak Proudfit

**Affiliations:** ^1^Department of Psychology, Center for Mind and Brain, University of CaliforniaDavis, Davis, CA, USA; ^2^Department of Psychology, Stony Brook UniversityStony Brook, NY, USA

**Keywords:** anxiety, attentional bias, dot probe, ERPs, IAPS, N2pc, reliability, threat

## Abstract

The dot-probe task is often considered a gold standard in the field for investigating attentional bias to threat. However, serious issues with the task have been raised. Specifically, a number of studies have demonstrated that the traditional reaction time (RT) measure of attentional bias to threat in the dot-probe task has poor internal reliability and poor test-retest reliability. In addition, although threatening stimuli capture attention in other paradigms, attentional bias to threat has not usually been found in typical research participants in the dot-probe task. However, when attention is measured in the dot-probe task with the N2pc component of the event-related potential waveform, substantial attentional orienting to threat is observed, and the internal reliability is moderate. To provide a rigorous comparison of the reliability of this N2pc measure and the conventional behavioral measure, as well as to examine the relationship of these measures to anxiety, the present study examined the N2pc in conjunction with RT in the dot-probe task in a large sample of participants (*N* = 96). As in previous studies, RT showed no bias to threatening images across the sample and exhibited poor internal reliability. Moreover, this measure did not relate to trait anxiety. By contrast, the N2pc revealed a significant initial shift of attention to threat, and this measure was internally reliable. However, the N2pc was not correlated with trait anxiety, indicating that it does not provide a meaningful index of individual differences in anxiety in the dot-probe task. Together, these results indicate a serious need to develop new tasks and methods to more reliably investigate attentional bias to threat and its relationship to anxiety in both clinical and non-clinical populations.

## INTRODUCTION

Threatening stimuli convey important information about the surrounding environment and are thought to automatically capture attention (e.g., LeDoux, 1996; Eastwood et al., 2001; Öhman et al., 2001a). The preferential allocation of attention to threatening stimuli over emotionally neutral stimuli, typically termed an *attentional bias* to threat, has become an important topic of investigation in the fields of affective and clinical science. In particular, abnormal allocation of attention to threat is thought to play a key role in anxiety disorders, providing a possible mechanism for distinguishing between normal and abnormal responses to threatening information (Beck, 1976; Williams et al., 1988; Mathews, 1990; Eysenck, 1992; Mathews and MacLeod, 2002; Bar-Haim et al., 2007; Cisler and Koster, 2010).

The dot-probe task, developed by MacLeod et al. (1986), is considered a gold standard in the field for investigating attentional bias to threatening stimuli. In this task, a threatening stimulus and a neutral stimulus are presented simultaneously in different spatial locations (e.g., one to the left visual field and one to the right visual field), followed by the presentation of a target item at one of the cued locations. Reaction times (RTs) to targets that appear at the prior location of the threatening stimulus (i.e., threat-congruent trials) are compared with RTs to targets that appear at the prior location of the neutral stimulus (i.e., threat-incongruent trials); faster responses on threat-congruent trials are interpreted as evidence of an attentional bias to the location of the threatening stimulus.

The dot-probe task has been used in hundreds of studies over the past three decades to investigate attention to threat-related stimuli in typical individuals and in clinically and non-clinically anxious individuals (see Bar-Haim et al., 2007 for a review). However, serious concerns with the dot-probe task have been raised. For example, although typical research participants appear to allocate attention to threatening information in the context of a variety of *other* tasks and measures (see MacNamara et al., 2013 for a review), there is no evidence of an attentional bias to threat in typical individuals using RT measures derived from the dot-probe task (see Bar-Haim et al., 2007 for a review). The dot-probe task almost uniquely suggests that normative individuals do *not* attend to threat. By contrast, an attentional bias to threat has been found in the dot-probe task among clinically and non-clinically anxious individuals (see Bar-Haim et al., 2007 for a review). However, even these results vary, with some studies failing to find a bias to threat in anxious populations (see, for example, Broadbent and Broadbent, 1988; Bradley et al., 1997, 2000; Mogg et al., 1997, 2000a,b; Mogg and Bradley, 1999; Yiend and Mathews, 2001; Pineles and Mineka, 2005). Indeed, a recent study found that anxious individuals exhibit a range of threat biases in the dot-probe task, including a bias to threat, a bias away from threat, or a combination of bias to and away from threat that depends on the specific type of threatening images examined (Zvielli et al., 2014).

One possible reason for such discrepant findings in the literature as well as the failure to find a bias to threat among typical individuals may be the poor psychometric properties of the RT measure derived from the dot-probe task. Specifically, a number of studies have collectively demonstrated that the traditional RT measure of attentional bias to threat used in the dot-probe task has both poor test-retest reliability (Schmukle, 2005; Staugaard, 2009) and poor internal reliability (Schmukle, 2005; Staugaard, 2009; Waechter et al., 2013; Kappenman et al., 2014; however, see Bar-Haim et al., 2010 for one contradictory finding). Indeed, the first study to demonstrate poor reliability of the RT-based measure of attentional bias to threat in the dot-probe task was published nearly a decade ago (Schmukle, 2005); however, the majority of dot-probe studies published since then do not provide a quantification of the internal consistency of the RT bias measure.

Internal consistency can be derived easily by computing split-half reliability (for example, by computing the correlation between the RT-based measure of threat bias derived from odd- versus even-numbered trials). This produces a measure of internal reliability—the degree to which RT bias to threat is consistent across the task *within* an individual. A number of studies have found poor internal reliability for the RT-based measure of attentional bias to threat using different versions of the dot-probe task, including the original version (Schmukle, 2005; Staugaard, 2009) and more recent modifications of the task (Schmukle, 2005; Staugaard, 2009; Waechter et al., 2013; Kappenman et al., 2014), and across different types of threat stimuli, including words (Schmukle, 2005), faces (Staugaard, 2009; Waechter et al., 2013), and complex images (Schmukle, 2005; Kappenman et al., 2014).

The fact that the RT measure of attentional bias to threat in the dot-probe task has poor internal reliability limits its validity: a measure cannot be valid unless it is reliable. Moreover, the internal reliability of a measure places an upper bound on its ability to correlate with another measure. Therefore, poor internal reliability of the RT-based measure of threat bias in the dot-probe task limits its ability to correlate with another measure, such as anxiety.

Recently, event-related potentials (ERPs) have been used in conjunction with RT measures to examine the time course of attention to threat in the dot-probe task with millisecond resolution (Kappenman et al., 2014). In contrast to behavioral measures, which reflect the combined effects of a sequence of many distinct neural processes, ERPs provide a continuous measure of processing and can therefore show how the allocation of attention unfolds over the course of a trial. In contrast to RT findings, ERPs have revealed an initial shift of visual attention to the threatening stimulus in typical individuals in the dot-probe task, as measured with the N2pc component (Kappenman et al., 2014), described in greater detail below. In other words, this ERP measure showed that typical individuals do indeed allocate attention to threat in the dot-probe task, despite the fact that RT measures (in this and many other tasks) showed no bias for threat in these individuals. That is, even within the same task and individuals, ERPs *but not behavioral measures* suggested that attention was biased to threatening stimuli in typical individuals. Moreover, this ERP measure of attention to threat was internally reliable, whereas the RT measure of threat bias exhibited poor internal reliability (Kappenman et al., 2014).

One reason the N2pc was able to capture an attentional bias to threat that was not evident in behavior is likely related to the timing of the measures relative to the events in the task. That is, the N2pc component was present from approximately 150–250 ms after the onset of the images, whereas the behavioral response occurred several hundred milliseconds later (after the presentation of the target item). Given that covert attention can shift rapidly between locations (in as little as 50–100 ms; Müller and Rabbit, 1989), ample time was provided for attention to shift away from the location of the threatening stimulus prior to the onset of the target. This was further supported in our previous study by the absence of sustained engagement with the threatening stimulus subsequent to the initial shift of attention (Kappenman et al., 2014). Specifically, our previous study found an N2pc to the threatening stimulus but no late positive potential (LPP)—an ERP index of sustained engagement with emotional images (see Hajcak et al., 2012 for a review of the LPP). Thus, the shift of attention to the threatening image had already terminated when the target appeared, which explains why the behavioral response to the target did not show evidence of an attentional bias to the threatening image location.

The present study extended this work by examining the relationship between ERP and behavioral measures of attentional bias to threat with individual differences in anxiety across a large sample (*N* = 96) of participants. The primary goal of this study was to determine whether the more internally reliable N2pc component might provide a better index of individual differences in anxiety—specifically, in contrast to the internally *un*reliable RT-based measure that has been the primary focus of the attentional bias literature.

We focused on the N2pc component, which is a negative-going potential at posterior electrode sites contralateral to the location of an attended item. This component has been well validated and has been used to index covert visual attention in cognitive psychology for over 25 years (see Luck, 2012 for a review), and more recently, to examine the allocation of attention to emotional stimuli (Eimer and Kiss, 2007; Fox et al., 2008; Buodo et al., 2010; Brosch et al., 2011; Shaw et al., 2011; Ikeda et al., 2013; Weymar et al., 2013; Grimshaw et al., 2014; Kappenman et al., 2014). We examined the N2pc in conjunction with the traditional RT measure of threat bias in the dot-probe task, investigating both the internal reliability of these measures and how they correlate with individual differences in trait-level anxiety. To ensure that we could distinguish anxiety from depression—which are often comorbid but may show distinct patterns of results in the dot-probe task (see Bar-Haim et al., 2007)—we used the Mood and Anxiety Symptom Questionnaire (MASQ; Watson and Clark, 1991; Watson and McKee, 1996) to separately measure facets of anxiety and depression in our sample. In addition, to maximize the potential for the task-irrelevant threatening stimuli to capture attention, we used complex threatening images from the International Affective Picture System (IAPS; Lang et al., 2008), which may be stronger elicitors of emotion than the emotional faces often used in dot-probe studies (Britton et al., 2006).

In line with previous studies, we predicted that we would find no evidence of an attentional bias to threat on average across the sample of participants in the present study using the RT-based measure of threat bias, and further, that this measure would have poor internal reliability. Poor internal reliability for the RT-based measure of threat bias would severely restrict the ability of this measure to correlate with any of our other measures, and therefore we predicted that the RT-based measure of threat bias would not meaningfully correlate with self-reported anxiety or depression. By contrast, we predicted that the N2pc would provide evidence of an initial shift of attention to threatening images across the sample, and that this measure would show moderate reliability, replicating our previous findings (Kappenman et al., 2014). Finally, we tentatively predicted that the N2pc would correlate with self-reported anxiety.

## MATERIALS AND METHODS

### PARTICIPANTS

One hundred and eleven undergraduate students between the ages of 18 and 30 were tested. In our research with typical young adults, participants are always excluded if they exhibit EEG artifacts on more than 25% of trials. Fifteen participants were excluded for this reason, leaving 96 participants (50 female, 46 male; Mean age = 20.54, SD = 2.34, Range 18–29); all analyses reflect this final sample. The study was approved by the University of California, Davis Institutional Review Board (IRB), and participants received monetary compensation.

### QUESTIONNAIRES

Prior to the start of the task, participants completed the MASQ, Short Form (Watson and Clark, 1991; Watson and McKee, 1996). The MASQ is a 62-item self-report measure consisting of four subscales, two that index anxiety symptoms, including “Anxious Arousal” (17 items) and “General Distress–Anxiety Symptoms” (11 items), and two that index depressive symptoms, including “Anhedonic Depression” (22 items) and “General Distress–Depressive Symptoms” (12 items). Participants are asked to indicate how much each item describes how they have felt “during the past week, including today” using a 5-point scale ranging from 1 (“Not at All”) to 5 (“Extremely”).

### STIMULI AND TASK

The stimuli were 50 neutral and 50 threatening images selected from the IAPS images^1^. Neutral images included pictures of buildings, household objects, and people with neutral facial expressions. Threatening images included pictures of animals attacking the viewer, assault and abduction scenes, and pictures of guns.

**FIGURE 1:**
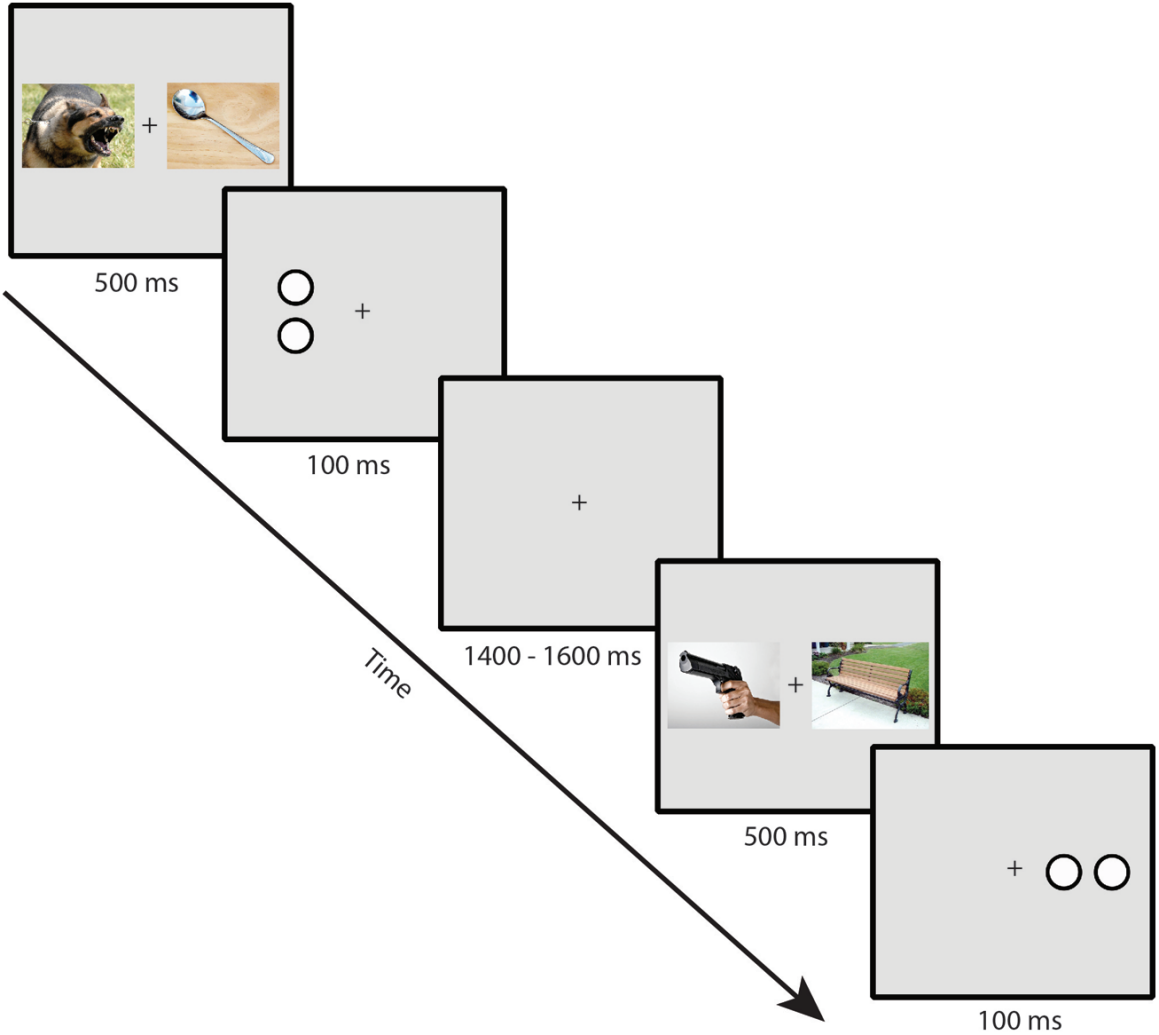
**Example trial sequence in the dot-probe task (note that stimuli are not to scale; see text for actual sizes used in the task)**.

Participants performed a dot-probe task. Example trial sequences are presented in **Figure 1**. Stimuli were presented on a gray background with a continuously visible fixation cross using a CRT monitor viewed at a distance of 70 cm. On each trial, a pair of IAPS images was presented for 500 ms, one image to the left and one image to the right of a continuously visible central black fixation cross. Each image in a pair subtended 10 × 7.3∘ of visual angle and was centered 6.2∘ to the left or right of the fixation cross. Immediately following the offset of the images, a target composed of either two horizontally or vertically arranged white dots outlined in black (each dot subtending 0.75∘ × 0.75∘ and separated by 0.15∘) was presented for 100 ms, centered in the location of one of the previously presented images. Participants made a button press using the index or middle finger of the dominant hand to indicate whether the target item was a pair of vertically or horizontally arranged dots. A jittered intertrial interval of 1400–1600 ms (rectangular distribution) with a blank screen occurred following the offset of the target. Participants were told that the images were irrelevant to the task and were instructed to respond to the targets as quickly and accurately as possible. To ensure that eye movement artifacts would not contaminate the EEG recordings and influence measurement of the N2pc, participants were instructed to maintain eye fixation in the center of the screen throughout the trial (see Luck, 2014).

The threat image appeared with equal probability on the left and right sides, as did the target, but the threat and target locations were independently randomized. The target orientation was equally likely to be horizontal or vertical, and this was randomized independently of the other variables. The combinations of threat location, target location, and target orientation were presented in an unpredictable order. Participants completed a total of 360 trials. Short self-paced breaks were provided every 40 trials.

### ELECTROENCEPHALOGRAPHIC RECORDING AND DATA PROCESSING

The continuous EEG was recorded using a Biosemi ActiveTwo recording system (Biosemi B.V., Amsterdam, the Netherlands). The electrodes were mounted in an elastic cap using a subset of the International 10/20 System sites (FP1, FP2, F3, F4, F7, F8, C3, C4, T7, T8, P1, P2, P3, P4, P5, P6, P7, P8, P9, P10, PO3, PO4, PO7, PO8, O1, O2, Fz, Cz, Pz, POz, Oz, and Iz). A common mode sense (CMS) electrode was located at site FC1, with a driven right leg (DRL) electrode located at site FC2. The horizontal electrooculogram (EOG) was recorded from electrodes placed lateral to the external canthi and was used to detect horizontal eye movements; the vertical EOG was recorded from electrodes placed above and below the right eye and was used to detect eyeblinks and vertical eye movements. The EEG and EOG were low-pass filtered using a fifth order sinc filter with a half-power cutoff at 204.8 Hz and digitized at 1024 Hz with 24 bits of resolution. The single-ended EEG signals were converted to differential signals oﬄine, referenced to the average of P9 and P10 (located adjacent to the mastoids).

Signal processing and analysis was performed in Matlab using EEGLAB toolbox (Delorme and Makeig, 2004) and ERPLAB toolbox (Lopez-Calderon and Luck, 2014). The EEG was high-pass filtered with a cut-off of 0.1 Hz (non-causal Butterworth impulse response function, half-amplitude cut-off, 12 dB/oct roll-off). Portions of EEG containing large muscle artifacts or extreme voltage offsets (identified by visual inspection) were removed. Independent component analysis (ICA) was then performed for each subject to identify and remove components that were clearly associated with eyeblink activity, as assessed by visual inspection of the waveforms and the scalp distributions of the components (Jung et al., 2000). The ICA-corrected EEG data were segmented for each trial beginning 200 ms prior to the onset of the images and continuing for 500 ms. Baseline correction was performed using the 200 ms prior to the onset of the images. Segments of data containing artifacts were removed by means of semi-automated ERPLAB algorithms, including eye movements larger than 0.1∘ of visual angle that were detected using the step function described by Luck (2014). Trials with incorrect behavioral responses or RTs of <200 or >1000 ms (relative to probe onset) were excluded from all analyses.

Reaction time was defined as the time of the button press relative to the onset of the target item on correct trials only; RTs were averaged separately for each condition. Accuracy was calculated as the percentage of correct trials per condition.

To determine whether attention was preferentially allocated to the threatening image, we isolated the N2pc time-locked to the onset of the image pairs at posterior electrode sites (P7 and P8, where the N2pc is typically maximal; Luck, 2012), relative to the location of the threatening image. Specifically, we first created separate waveforms for the hemisphere that was contralateral to the threatening image (i.e., left hemisphere electrode sites for right-side threatening images, and right hemisphere electrode sites for left-side threatening images) and the hemisphere that was ipsilateral to the threatening image (i.e., right hemisphere electrode sites for right-side threatening images, and left hemisphere electrode sites for left-side threatening images). We then created a contralateral-minus-ipsilateral difference waveform, and the N2pc was measured from the resulting difference wave in each subject. The mean amplitude of the N2pc was measured using an a priori time window of 175–225 ms following the onset of the image pairs, as in our previous study (see Kappenman et al., 2014).

Pearson’s correlations were used to examine the relationship among measures^2^. Split-half reliability analyses were conducted by computing correlations of the averages of odd-numbered trials and even-numbered trials. All split-half reliability analyses were corrected for length using the Spearman–Brown formula (Anastasia and Urbina, 1997); all reported values reflect this correction.

## RESULTS

### BEHAVIOR

Mean RTs and mean accuracy (percent correct) are shown in **Table 1**. Participants were just as accurate for targets that replaced threatening images compared to targets that replaced neutral images [*t*(95) = 0.128, *p* > 0.898]. Consistent with previous studies, no significant RT difference was found between targets that replaced threatening images (threat-congruent trials) and targets that replaced neutral images (threat-incongruent trials) across the sample of participants [*t*(95) = 1.01, *p* > 0.314]. In other words, the sample as a whole demonstrated no evidence of bias toward or away from threat with RT, replicating many previous findings in the literature. We also examined the internal reliability of the RT bias measure (the difference between RT on threat-incongruent and threat-congruent trials); mean values for odd- and even- numbered trials are shown in **Table 2**. The threat bias measure derived from RT was uncorrelated between odd- and even-numbered trials (*r* = 0.030, *p* > 0.772), indicating poor internal reliability for this measure.

**Table 1:** Behavioral measures (SD in parentheses).

Trial type	Accuracy (Correct)	Mean RT (ms)
All trials	95.57 (3.7)	527.93 (73.7)
Threat-congruent	95.58 (3.8)	529.15 (75.6)
Threat-incongruent	95.55 (3.9)	526.89 (74.2)

**Table 2:** Split-half reliability measures (SD in parentheses).

Trial type	Mean RT-bias (ms)	N2pc mean amplitude	(V)
Odd-numbered	2.26 (16.35)	-0.66 (0.95)
Even-numbered	-0.08 (13.89)	-0.66 (1.03)

The logic of traditional null hypothesis statistical testing does not make it possible to conclude from the lack of statistically significant differences between threat-congruent and threat-incongruent trials that these conditions yielded equivalent RT or equivalent accuracy. However, it is possible to convert the *t* values from these analyses into *Bayes factor* values, which indicate the relative likelihood of the null and alternative hypotheses (Rouder et al., 2009). When we computed the Bayes factor for RT in the present study (using the calculator at ), we found that the null hypothesis was 7.5 times more likely to be true than the alternative hypothesis of a difference in RT between threat-congruent and threat-incongruent trials. Similarly, the null hypothesis was 12.3 times more likely to be true than the alternative hypothesis for accuracy. To provide a sense of the magnitude of these Bayes factor values, we also computed the Bayes factor that we would have obtained with this sample size if we had found a just-barely significant difference [i.e., *t*(95) = 2.0, *p* = 0.049] between threat-congruent and threat-incongruent trials. If we had obtained this *t* value, the corresponding Bayes factor value would have been 1.8, meaning that the alternative hypothesis would have been only 1.8 times more likely to be true than the null hypothesis. By comparison, the Bayes factor values of 7.8 and 12.3 that we actually found in favor of the null hypothesis are quite substantial.

### N2pc

**FIGURE 2:**
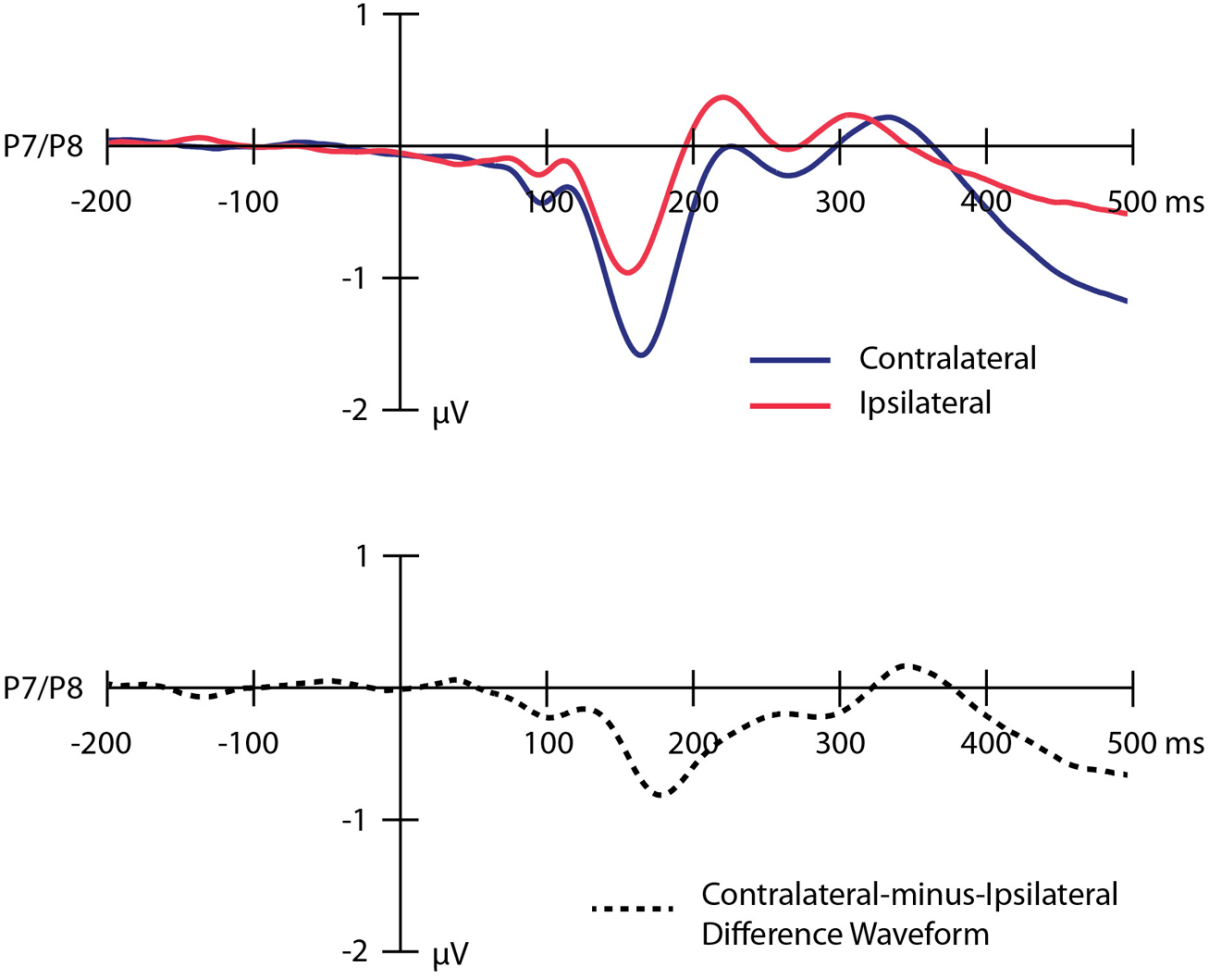
**Grand average event-related potential waveforms time-locked to the onset of the images collapsed across the P7 and P8 electrode sites.** The top panel shows the waveforms contralateral to the location of the threatening image (dark blue line), ipsilateral to the location of the threatening image (light red line). The bottom panel shows the contralateral-minus-ipsilateral difference waveform (dotted black line). A digital low-pass filter was applied oﬄine before plotting the waveforms shown here (Butterworth impulse response function, half-amplitude cutoff = 15.0 Hz, 12dB/oct roll-off).

Grand average ERP waveforms time-locked to the onset of the IAPS images and collapsed across the P7 and P8 electrode sites are presented in **Figure 2**. The top panel overlays the waveforms contralateral to the location of the threatening image (dark blue line) and ipsilateral to the location of the threatening image (light red line). The bottom panel shows the contralateral-minus-ipsilateral difference waveform (dotted black line). Analyses revealed a significant N2pc (*M* = -0.66 μV, SD = 0.82) in the contralateral-minus-ipsilateral difference waveform [*t*(95) = 7.96, *p* < 0.001], reflecting a shift of covert visual attention in the direction of the threatening image following the onset of the image pair. The corresponding Bayes factor value indicated that the hypothesis of a real difference between the contralateral and ipsilateral voltages was over 1000 times more likely to be true than the null hypothesis.

Mean amplitude values for the N2pc on odd- and even-numbered trials are shown in **Table 2**. The amplitude of the N2pc on odd- versus even-numbered trials was moderately correlated (*r* = 0.515, *p* < 0.001), indicating that the N2pc was somewhat internally reliable (and much more reliable than the behavioral measures). These findings replicate our previous study conducted with participants from a different university (see Kappenman et al., 2014).

### CORRELATIONS

**FIGURE 3:**
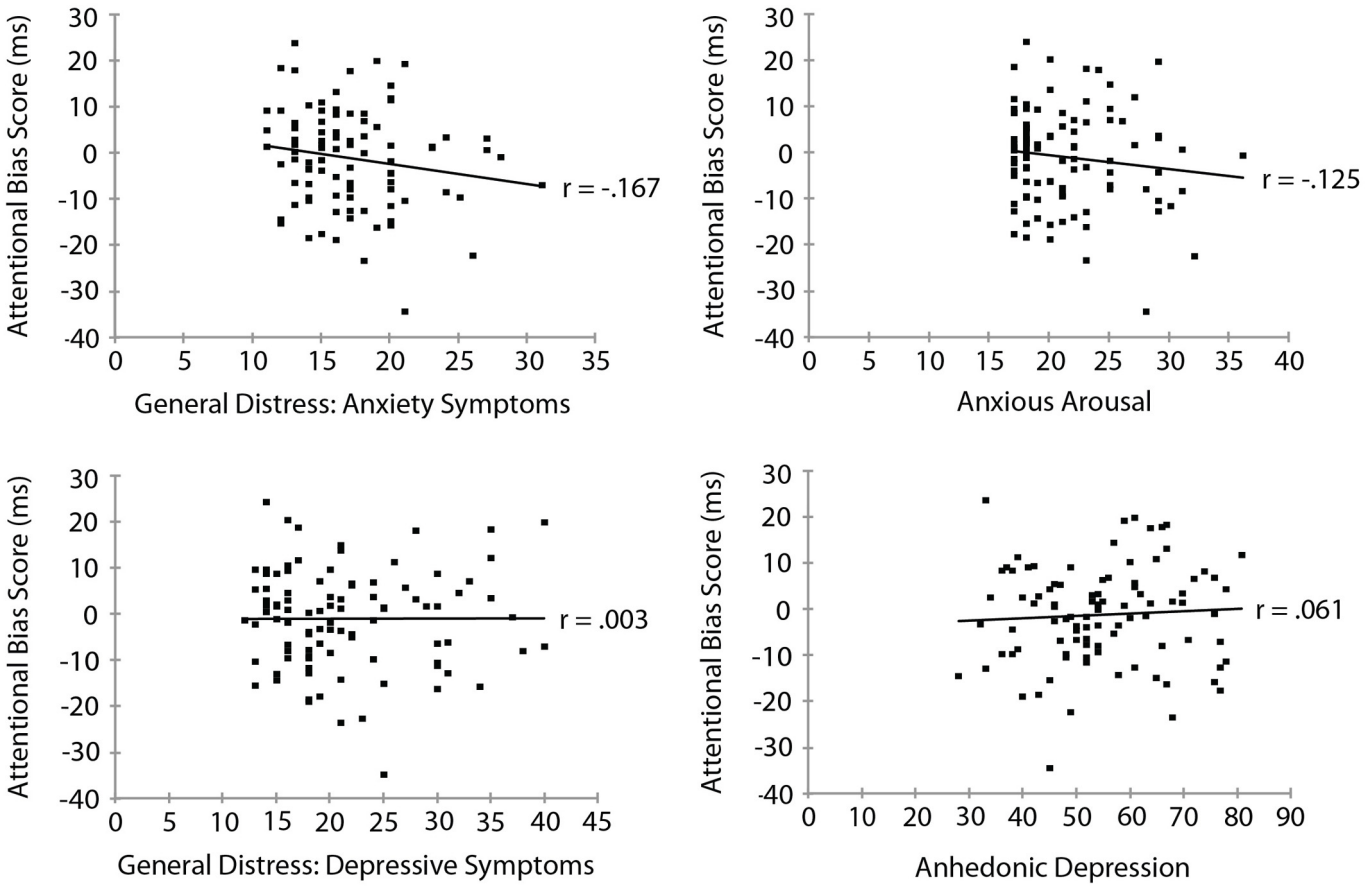
**Reaction time measure of attentional bias (threat-incongruent minus threat-congruent) as a function of MASQ subscale scores**.

**FIGURE 4:**
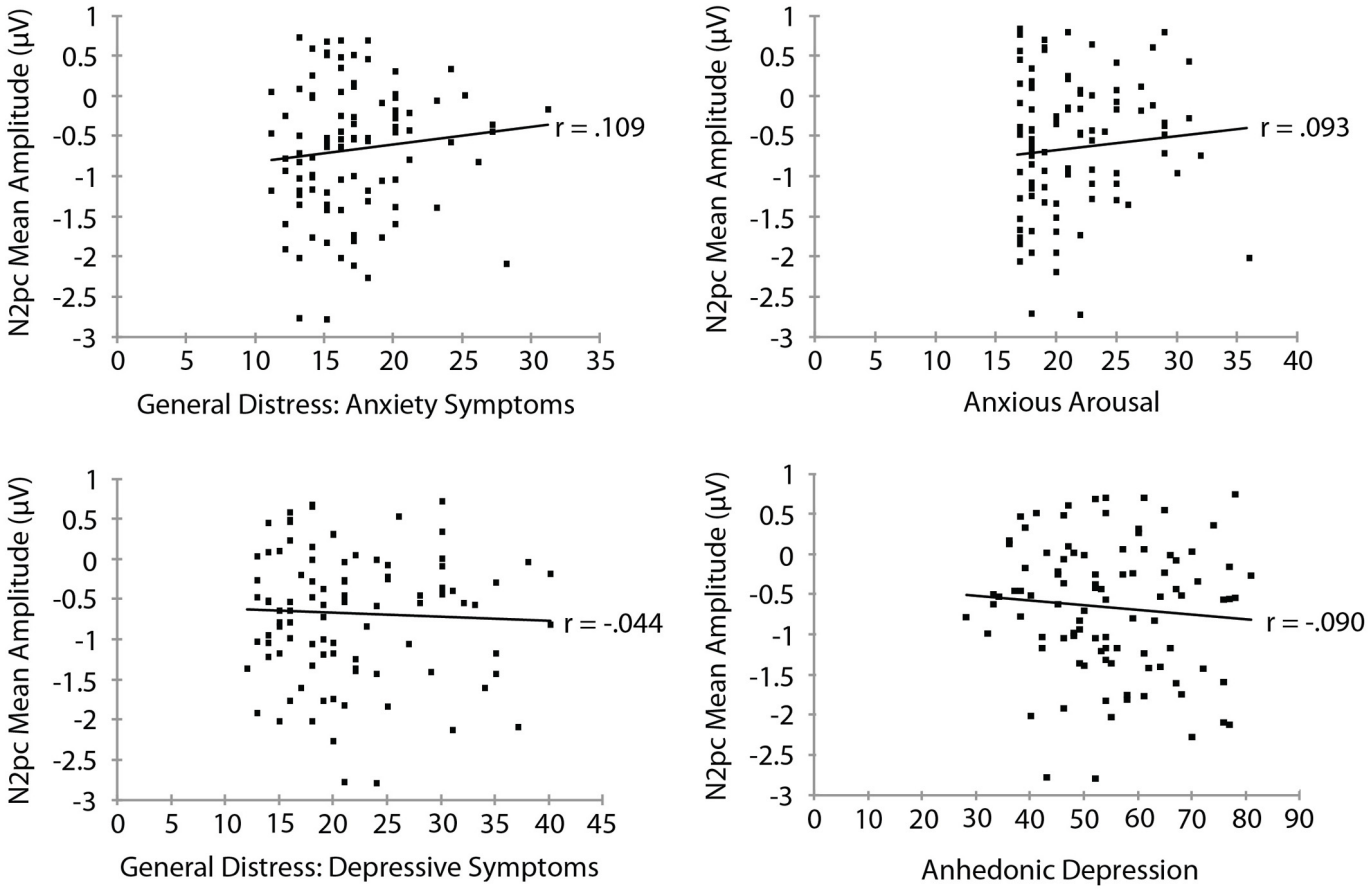
**Mean amplitude of the N2pc as a function of Mood and Anxiety Symptom Questionnaire subscale scores**.

Mood and Anxiety Symptom Questionnaire subscale measures are summarized in **Table 3**. To examine the relationship between threat bias and anxiety, we correlated each of the MASQ subscale scores separately with each of the measures of threat bias. The RT measure of attentional bias as a function of each of the MASQ subscale scores is shown in **Figure 3**. No significant correlation was found between the RT measure of threat bias and any of the MASQ subscales, including the anxiety and depression subscales (all *p*s > 0.10). Note that the non-significant correlations between the RT-based measure of threat bias and anxiety subscales were *negative* correlations, indicating that higher levels of anxiety were (non-significantly) associated with a *smaller* attentional bias to threat. This is the opposite of what would be predicted. The mean amplitude of the N2pc as a function of each of the MASQ subscale scores is shown in **Figure 4**. Despite the significant internal reliability of the N2pc, no significant correlation was obtained between the N2pc and the MASQ subscales (all *p*s > 0.29).

**Table 3:** MASQ measures (SD in parentheses).

Subscale	Mean score
General Distress: Anxiety symptoms	17.02 (4.0)
Anxious arousal	21.42 (4.4)
General Distress: Depressive symptoms	21.52 (7.0)
Anhedonic depression	54.49 (12.6)

We also examined whether attention to threat indexed by the N2pc was related to behavior by correlating the difference in RT on threat-incongruent and threat-congruent trials with the amplitude of the N2pc. No relationship between the N2pc and the RT measure of threat bias was found (*r* = -0.079, *p* > 0.445).

## DISCUSSION

The present study examined the relationship between behavioral and ERP measures of attentional bias to threat in a dot-probe task and measures of trait anxiety in a large sample of participants. In line with previous dot probe studies, we found no evidence of an attentional bias to threat across the sample using the traditional RT measure of threat bias in this task (i.e., the difference in RT on threat-incongruent and threat-congruent trials). In addition, the RT-based measure of threat bias was not internally reliable—RT-based measure of bias derived from odd and even trials were uncorrelated—a finding consistent with many previous studies (Schmukle, 2005; Staugaard, 2009; Waechter et al., 2013; Kappenman et al., 2014). Insofar as internal reliability limits both the validity of a measure and the ability of a measure to correlate with another trait-like measure, the RT-based measure of threat bias in this task was not an appropriate measure of individual differences in anxiety. Indeed, even if a significant relationship between RT-based threat bias and anxiety had emerged in the present study, the lack of internal reliability for the RT measure would have made the correlation uninterpretable.

Although we found no evidence of an attentional bias to threat using RT, ERPs revealed that there was an attentional bias to threat in our sample, as reflected by an N2pc to the location of the threatening stimulus. In addition, the N2pc showed highly significant (but not impressive) internal reliability, replicating the results of our previous study (Kappenman et al., 2014). These results showed that individuals are somewhat consistent in the degree to which they exhibit an electrocortically measured attentional bias to threatening stimuli in the dot-probe task. This is in direct contrast to the results obtained with behavior, which showed (1) no evidence of an attentional bias to threat and (2) no consistency within an individual.

One likely reason the N2pc was able to capture an attentional bias to threat that was not evident in behavior is that the N2pc is a direct measure of attention time-locked to the onset of the threatening stimulus. By contrast, the behavioral response is made to a separate target stimulus presented hundreds of milliseconds after the initial onset of the threatening stimulus. Given that covert attention can shift rapidly between locations, it is likely that although attention was initially allocated to the threatening image (as reflected by the N2pc), attention shifted away from the threatening stimulus location prior to the onset of the target. This was supported by our previous study, which found an initial shift of attention to threatening images but no evidence of sustained engagement with threat subsequent to the initial allocation of attention (Kappenman et al., 2014). A number of studies have found similar dissociations between the N2pc and RT-based measures (Fenker et al., 2009; Ikeda et al., 2013; Kiss et al., 2013). It is possible that a modification of the timing of the events in the dot-probe task—for example, by presenting the target during the time of the initial shift of attention reflected by the N2pc—might provide a way of capturing the initial shift of attention to threat with behavioral measures.

Despite the modest internal reliability of the N2pc measure of threat bias and the large sample size used in the present study, we found no evidence of a relationship between anxiety and the amplitude of the N2pc. A similar result was obtained in a recent study, which found an N2pc to angry faces but no relationship between the N2pc and self-reported levels of anxiety in a non-clinical sample (Grimshaw et al., 2014). Thus, the N2pc may provide a more reliable marker of attentional bias to threat than RT in the dot-probe task, but it appears to be unrelated to individual differences in anxiety in non-clinical samples.

It is important to note that thus far dot-probe studies examining the N2pc have all used inherently threatening stimuli, which differ on low-level physical stimulus properties (e.g., luminance, spatial frequency, etc.). These low-level physical stimulus differences may influence the amplitude of early sensory-related ERP components, including the N2pc (see Luck and Kappenman, 2012; Luck, 2012, 2014). Therefore, it is impossible to determine on the basis of existing studies whether the initial shift of attention to threat in the dot-probe task indexed by the N2pc is specifically related to the *emotional* content of the stimuli, differences in low-level physical stimulus properties that are naturally conflated with emotional content, or a combination of both of these factors. Indeed, it may not be fully possible to separate out emotional content from differences in low-level physical stimulus properties (for example, see Larson et al., 2009). An important direction for future research could be to examine attentional bias to *conditioned* threat stimuli, which would provide a way of fully controlling for physical stimulus properties across participants. If the N2pc is partially determined by low-level physical stimulus differences and not by the emotional content of the images *per se*, this might help explain why this early signature of attentional bias to threat does not correlate with anxiety.

Together, the findings of the present study call into question the appropriateness of the dot-probe task as the primary method for examining attentional bias to threat across populations. Specifically, multiple studies have demonstrated that the RT-based measure of threat bias in the dot-probe task is unreliable, both in terms of internal reliability (Schmukle, 2005; Staugaard, 2009; Waechter et al., 2013; Kappenman et al., 2014) and test-retest reliability (Schmukle, 2005; Staugaard, 2009). However, this RT-based measure of attentional bias to threat in the dot-probe task has remained the primary measure used in the field, and the majority of dot probe studies still do not quantify the internal consistency of threat bias measures. This makes it difficult to interpret the results of studies, especially studies that include correlations between RT-based threat bias and other trait-based measures, such as anxiety.

The fact that typical individuals fail to show an attentional bias to threat using behavioral measures in the dot-probe task is also concerning in light of clear evidence that these individuals do exhibit biased attention to threat both in the dot-probe task (revealed by ERPs in the present study; also see Kappenman et al., 2014), and in other tasks (with RT and ERP measures; see MacNamara et al., 2013 for a review). In other words, the RT-based measure of threat bias in the dot-probe task is not capturing an attentional bias to threat among individuals who clearly show such a bias using alternative measures. This could directly impact the ability of this measure to elucidate differences in normal and abnormal reactions to threatening stimuli.

In contrast to the RT-based threat measure, the N2pc did suggest an attentional bias to threat in the dot-probe task; however, the internal reliability of the N2pc was somewhat unimpressive—suggesting that this measure too may not be an ideal individual difference measure of the initial allocation of attention to threatening stimuli in this task. This means that after decades of research we still lack a measure of attentional bias to threat in the dot-probe task that can reliably index individual differences in anxiety. Note, however, that the reliability of the N2pc depends on the number of trials being averaged together, so the reliability could be increased by using a much larger number of trials per subject—a possible direction for future research.

Collectively, these data suggest that it is time to develop new tasks and measures to index attentional bias to threatening information and assess the role of attentional bias to threat in anxiety. An alternative approach to continued reliance on the dot-probe task might involve adapting other tasks designed to measure attentional processes in the cognitive psychology literature for use in emotion and anxiety. For example, visual search appears to provide a promising alternative, including a long history of use in the basic science literature and easy integration with ERP and eye-tracking measures. Furthermore, visual search has been adopted already with modest success in the emotion, anxiety, and depression literatures (see, for example, Öhman et al., 2001b; Wisco et al., 2012; Lundqvist et al., 2014). However, note that one study found relatively low reliability for behavioral bias measures derived from visual search in children 8–10 years of age (Brown et al., 2014). Combining neural measures with the development of alternative tasks may provide the most promising avenue for future research to obtain conclusive evidence about the role of attentional bias to threat in anxiety. As a field, it is time to move beyond the dot-probe task as our primary paradigm.

## Conflict of Interest Statement

The authors declare that the research was conducted in the absence of any commercial or financial relationships that could be construed as a potential conflict of interest.

## Footnotes

1 Threatening IAPS images were: 1026, 1033, 1050, 1052, 1080, 1090, 1113, 1114, 1120, 1200, 1201, 1202, 1205, 1220, 1230, 1240, 1300, 1301, 1302, 1304, 1321, 1525, 1726, 1820, 1930, 1931, 1932, 2120, 2691, 2811, 3500, 6190, 6200, 6211, 6213, 6220, 6230, 6240, 6242, 6243, 6250.1, 6260, 6263, 6370, 6510, 6940, 9425, 9426, 9440, 6270.1. Neutral IAPS images were: 2102, 2191, 2383, 2384, 2411, 2745.1, 5390, 5395, 5500, 5530, 5731, 6150, 7000, 7001, 7002, 7004, 7006, 7009, 7010, 7012, 7018, 7019, 7020, 7021, 7025, 7026, 7031, 7032, 7033, 7035, 7036, 7037, 7040, 7041, 7042, 7056, 7057, 7059, 7060, 7061, 7080, 7081, 7090, 7150, 7175, 7211, 7233, 7512, 7546, 7547.

2 We also examined non-parametric (i.e., Spearman’s rank) correlations, which showed the same pattern of results.
